# Synthetic CT for the planning of MR-HIFU treatment of bone metastases in pelvic and femoral bones: a feasibility study

**DOI:** 10.1007/s00330-022-08568-y

**Published:** 2022-02-21

**Authors:** Beatrice Lena, Mateusz C. Florkow, Cyril J. Ferrer, Marijn van Stralen, Peter R. Seevinck, Evert-Jan P. A. Vonken, Martijn F. Boomsma, Derk J. Slotman, Max A. Viergever, Chrit T. W. Moonen, Clemens Bos, Lambertus W. Bartels

**Affiliations:** 1 Image Sciences Institute, Division of Imaging and Oncology, University Medical Center Utrecht, Utrecht University, Heidelberglaan 100, Q.02.4.45, 3584 CX Utrecht, The Netherlands; 2 Division of Imaging and Oncology, University Medical Center Utrecht, Utrecht University, Heidelberglaan, 100 3584 CX Utrecht, The Netherlands; 3 MRIguidance BV, Gildstraat 91-A, 3572 EL Utrecht, The Netherlands; 4 Division of Imaging and Oncology, Department of Radiology, University Medical Center Utrecht, Utrecht University, Heidelberglaan, 100 3584 CX Utrecht, The Netherlands; 5 Department of Radiology, Isala Hospital, Dokter van Heesweg 2, 8025 AB Zwolle, The Netherlands

**Keywords:** MRI, CT, Synthetic CT, High-intensity focused ultrasound ablation, Neoplasm metastasis

## Abstract

**Objectives:**

Visualization of the bone distribution is an important prerequisite for MRI-guided high-intensity focused ultrasound (MRI-HIFU) treatment planning of bone metastases. In this context, we evaluated MRI-based synthetic CT (sCT) imaging for the visualization of cortical bone.

**Methods:**

MR and CT images of nine patients with pelvic and femoral metastases were retrospectively analyzed in this study. The metastatic lesions were osteolytic, osteoblastic or mixed. sCT were generated from pre-treatment or treatment MR images using a UNet-like neural network. sCT was qualitatively and quantitatively compared to CT in the bone (pelvis or femur) containing the metastasis and in a region of interest placed on the metastasis itself, through mean absolute difference (MAD), mean difference (MD), Dice similarity coefficient (DSC), and root mean square surface distance (RMSD).

**Results:**

The dataset consisted of 3 osteolytic, 4 osteoblastic and 2 mixed metastases. For most patients, the general morphology of the bone was well represented in the sCT images and osteolytic, osteoblastic and mixed lesions could be discriminated. Despite an average timespan between MR and CT acquisitions of 61 days, in bone, the average (± standard deviation) MAD was 116 ± 26 HU, MD − 14 ± 66 HU, DSC 0.85 ± 0.05, and RMSD 2.05 ± 0.48 mm and, in the lesion, MAD was 132 ± 62 HU, MD − 31 ± 106 HU, DSC 0.75 ± 0.2, and RMSD 2.73 ± 2.28 mm.

**Conclusions:**

Synthetic CT images adequately depicted the cancellous and cortical bone distribution in the different lesion types, which shows its potential for MRI-HIFU treatment planning.

**Key Points:**

• *Synthetic computed tomography was able to depict bone distribution in metastatic lesions.*

• *Synthetic computed tomography images intrinsically aligned with treatment MR images may have the potential to facilitate MR-HIFU treatment planning of bone metastases, by combining visualization of soft tissues and cancellous and cortical bone.*

**Supplementary Information:**

The online version contains supplementary material available at 10.1007/s00330-022-08568-y.

## Introduction

Magnetic resonance imaging–guided high-intensity focused ultrasound (MRI-HIFU) has shown promising results for therapy in bones and joints [1–4]. Particularly, MRI-HIFU has shown potential for pain palliation in patients with bone metastases [5], where the suspected mechanism of action is the thermal ablation of the nerves that produce the pain [6–8].

The treatment volume may be limited to the superficial periosteum for palliative denervation, or it can also involve ablation of the deeper tumor tissue [2]. During treatment planning for pain palliation, adequate depiction of the cancellous and cortical bone in and around the lesion in treatment position is required for choosing a treatment strategy appropriate for the lesion type [9]. In case of tumor ablation, delineation of the lesion is also required. MRI is suitable to characterize soft tissues within or adjacent to the periosteum. However, computed tomography (CT) is better suited to expose cortical bone loss (in osteolytic lesions) or bone creation (in osteoblastic lesions) [10], inherent to bone metastases.

Currently, patient eligibility assessment for HIFU treatment and lesion characterization are done using CT scans, and occasionally pre-treatment MRI scans, acquired prior to HIFU treatment [2, 11]. These scans are also used on the treatment day to choose a suitable treatment strategy approach, depending on the integrity of the bone cortex [9, 12]. However, patient positions in pre-treatment and treatment images usually differ. In addition, pre-treatment images may have been acquired up to months prior to the MRI-HIFU treatment procedure, so clinically significant delay may exist between the scans.

To provide information on the cancellous and cortical bone distribution in and around the lesion in MRI-HIFU treatment position, strategies to register pre-treatment CT to MR images have been investigated. However, interscan registration was reported to be a complex time-consuming process and pathological changes over time were ignored [13].

In the last decade, the development of synthetic CT (sCT), i.e., deriving CT-like images from MRI scans, has enabled an MRI-based visualization of osseous tissues for radiotherapy [14] and orthopedic care [15–18]. In the MRI-HIFU context, such sCT images could be generated from MR images acquired during the treatment session to depict the bone distribution in the treatment position. Although sCT generation has been used for orthopedic purposes [17–19] and HIFU treatment planning [20], its ability to reconstruct bone blastic or lytic lesions is unknown.

We implemented a fast and automated method for sCT generation that enables the combined visualization of soft tissue and cortical bone. By qualitatively and quantitatively comparing sCT with CT, we investigated the potential of sCT images for visualizing cancellous and cortical bone in patients with bone metastases.

## Methods

### Patient data

Imaging data of nine patients were used retrospectively for this study. The patients were screened for palliative MRI-HIFU treatment of bone metastases at the radiology department of Isala Hospital (Zwolle, The Netherlands), between January 2019 and December 2019, and written informed consent for the use of their data for scientific research was obtained. All patients underwent radiotherapy before the MRI-HIFU treatment. For the purpose of this study, all data were anonymized.

### Acquisition parameters

All patients had a pre-treatment CT scan, available from radiotherapy planning. Acquisition parameters are shown in Table 1.

**Table 1:** Main imaging sequence parameters for pre-treatment CT and pre-treatment and treatment MR scans

CT	
CT scanner	iCT 256, Brilliance Big Bore or Ingenuity CT, Philips Healthcare
In-plane reconstructed pixel size (range)	[0.67–0.98] × [0.67–0.98] mm^2^
Slice spacing	3 mm
Tube voltage	120 kV
Exposure	[69–253] mAs
MR	
MR scanner	Achieva, Philips Healthcare
Type	Radiofrequency-spoiled T1-weighted multi-echo gradient-echo
Scan mode	3D
In-plane pixel size (range)	[0.92–1] × [0.92–1] mm^2^
Partition thickness	2 mm
TE1/TE2/TR	2.1/4.2/7 ms
Flip angle	10°
Number of slices	150
Acquisition duration	≈ 3 min

Values between square brackets indicate a range

*TE* echo time, *TR* repetition time

MRI scans were acquired on a 1.5-T scanner. Pre-treatment MRI scans were acquired with a 16-element coil with the patient in supine position. Treatment MRI scans were acquired with an integrated 2-element coil inside the HIFU tabletop (Sonalleve V2, Profound Medical) combined with an external 3-element pelvic coil. Treatment position was chosen to minimize the distance between the transducer and the lesion, with proper acoustic access to the lesion. In both MRI sessions, the same sequence was included. Acquisition parameters are shown in Table 1.

The average time between acquisition of the pre-treatment CT and the treatment MR was 61 days (range: [1; 165] days), whereas the average time between pre-treatment CT and pre-treatment MRI, which was available for four patients, was 31 days (range: [6; 104] days). If available, pre-treatment MR images were used for training and evaluation to minimize interscan positioning and pathophysiological differences. If not, treatment MR images were used (*n* = 5/9).

### Preprocessing

#### Segmentation

The pelvic and femoral bones were semi-automatically segmented on the MR and CT images using image processing software (Mimics Medical 21.0, Materialize). Lesion masks were semi-automatically created on the MR images by a radiologist, using segmentation software (ITK-Snap v3.8.0 [21]).

#### Registration

**Fig. 1:**
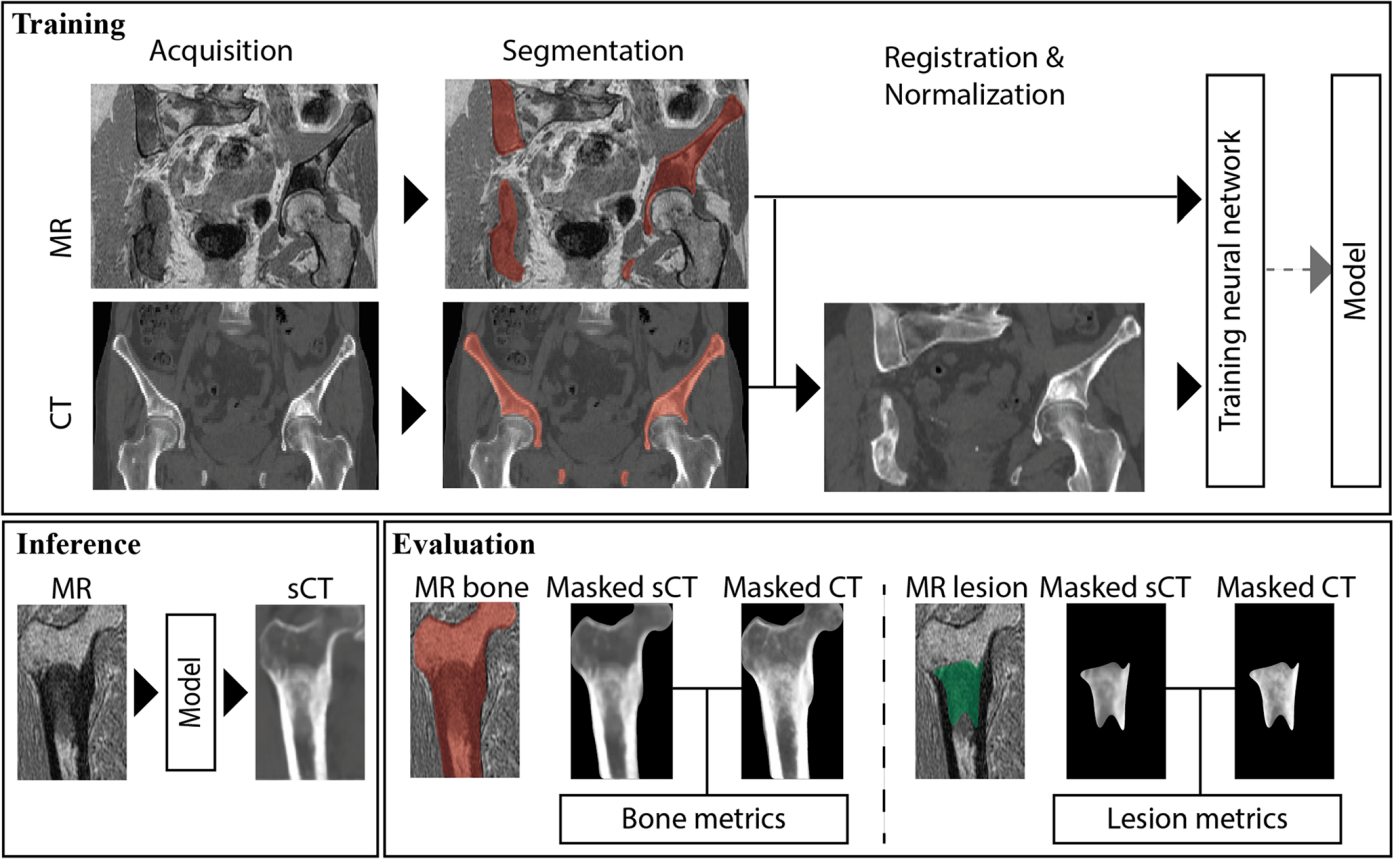
Schematic description of the approach. The bone containing the lesion was segmented on MRI and CT and used to register the CT to the MRI. Patches of 24 × 24 × 24 voxels were then extracted from the MRI and registered CT to train a synthetic CT (sCT) generation model. Once trained, the model was used to create a sCT of the bone of interest from a patient not seen during the training. The sCT was then evaluated in the bone of interest and the metastatic lesion

CT images were registered to the MR images in two steps. First, an iterative closest point algorithm [22] applied on the MR– and CT–based bone segmentations combined with a dual quaternion interpolation of the soft tissues initialized the registration [23]. Then, an image-based deformable registration was applied on the entire body contour, using open source registration software (Elastix [24]). Given the challenging registration task due to large differences in patients’ positioning between the scanning sessions (Fig. 1), this registration focused on matching the bones at the expense of the surrounding soft tissues. During registration, CT images were resampled to the MRI resolution using cubic B-spline interpolation. In the remainder of the paper, CT refers to the registered CT.

#### Normalization

MR intensities were clipped beyond the 95^th^ percentile to exclude the hyperintense signal from the fluid surrounding the transducer inside the HIFU tabletop and then linearly mapped to [− 1; 1]. CT intensities were linearly mapped from the Hounsfield unit range [− 1024; 3071] to [− 1; 1].

### Neural network

Synthetic CT images were generated by means of a 3D patch-based UNet-like neural network [25, 26], using an architecture and hyperparameters previously applied for sCT generation in the hip [27]. The neural network took as inputs MR images at two echo times and was trained to minimize the L1 distance between the CT and sCT at a learning rate of 10^−4^ using an Adam optimizer [28]. During the training, data were augmented to artificially increase the training set. Random flipping of the coronal and sagittal planes and random rotation between − 45 and 45° around the feet-head axis were applied to simulate the unconventional patient positioning.

Because of the small amount of data, the network was trained using leave-one-out cross-validation resulting in nine models. For each model, seven data sets were used for training, one for validation, and one as an independent test set. Training and testing were done on a GeForce RTX 2080 Ti (NVIDIA) graphics processing unit.

### Evaluation

To evaluate accuracy, sCT images were compared to CT images in the bone containing the metastasis and the metastasis itself, using the masks as segmented on the MR images. The metrics used for evaluation were the mean sCT-to-CT difference (MD), mean absolute difference (MAD), the Dice similarity coefficient (DSC) [29] and the sCT-to-CT root mean square surface distance (RMSD) [30]. For the analysis on the bone and the lesion, cancellous and cortical bone were extracted from the CT and sCT images, by means of a 150-HU [31, 32] threshold applied within the bone and the lesion masks, respectively. The DSC and the surface distance were computed between CT and sCT on the extracted bones. Particularly for the surface distance estimation, the lesion masks included a 3-cm margin around them. These methodological steps are summarized in Fig. 1.

## Results

The characteristics of the population are described in Table 2.

**Table 2:** Demographics and clinical characteristics of the patients

Patient	Patient sex	Primary tumor type	Metastasis type	Location of the metastasis	Days between CT scan and HIFU treatment
P1	Male	Bladder	Mixed	Pelvis	13
P2	Female	Breast	Mixed	Pelvis	35
P3	Female	Colon	Osteoblastic	Pelvis	157
P4	Male	Lung	Osteolytic	Femur	165
P5	Female	Bile duct	Osteolytic	Femur	10
P6	Male	Prostate	Osteoblastic	Femur	1
P7	Male	Prostate	Osteoblastic	Pelvis	1
P8	Male	Liver	Osteolytic	Pelvis	126
P9	Male	Prostate	Osteoblastic	Pelvis	3

Synthetic CT images were generated in 66 s from an MR image of matrix size 352 × 352 × 150. On average per patient, sCT images were generated in 80 s from the T1-w gradient-echo images.

Metrics for the quantitative comparison of CT and sCT for all patients are reported in Table 3 for the bone containing the lesion and in Table 4 for the lesion only. Average ± standard deviation voxelwise differences were as follows: MAD 116 ± 26 HU and MD: − 14 ± 66 HU in the bone and MAD: 132 ± 62 HU and MD: − 31 ± 106 HU. The MD in the bone of interest was overall negative, which indicates that bone intensities in sCT images were on average underestimated.

**Table 3:** Mean absolute difference (MAD), mean difference (MD), Dice similarity coefficient (DSC), and root mean square difference (RMSD) obtained for each patient between the sCT and CT in the bone containing the lesion. DSC and RMSD were obtained using a threshold of 150 Hounsfield units

P#	MAD (HU)	MD (HU)	DSC (1)	RMSD (mm)
P1	102	34	0.81	2.70
P2	146	23	0.86	2.54
P3	132	− 26	0.74	1.74
P4	96	− 47	0.85	1.38
P5	77	29	0.88	1.67
P6	154	119	0.90	1.90
P7	136	− 98	0.89	2.11
P8	109	87	0.9	1.76
P9	95	7	0.85	2.65

**Table 4:** Mean absolute difference (MAD), mean difference (MD), Dice similarity coefficient (DSC), and root mean square difference (RMSD) obtained for each patient between the sCT and CT in the metastatic lesion. DSC and RMSD were obtained using a threshold of 150 Hounsfield units. Patients are stratified per lesion type

Lesion type	P#	MAD (HU)	MD (HU)	DSC (1)	RMSD (mm)
Osteolytic	P4	188	100	0.74	1.42
	P5	27	12	0.77	0.75
	P8	108	− 51	0.60	3.87
Osteoblastic	P3	166	101	0.82	2.08
	P6	235	− 229	0.96	0.89
	P7	81	29	0.99	2.08
	P9	149	− 123	0.49	7.30
Mixed	P1	94	− 64	0.56	4.93
	P2	143	− 55	0.86	2.86

Despite voxelwise differences, the bone distribution was similar between the CT and sCT, as evidenced by an average DSC among patients of 0.85 ± 0.05 in the bone and of 0.75 ± 0.2 in the lesion.

The surface of the bone and the osseous tissues inside the lesion have been preserved in the sCT, as indicated by the sCT-to-CT surface distance analysis. The largest errors have been found close by and in the lesion: the average ± standard deviation RMSD equals 2.73 ± 2.28 mm in and around the lesion compared to 2.05 ± 0.48 mm in the entire bone. Three-dimensional bone renderings with overlays of sCT-to-CT surface distances obtained in the bone and in the lesion are available for all patients in Supplementary Material Figure S1.

**Fig. 2:**
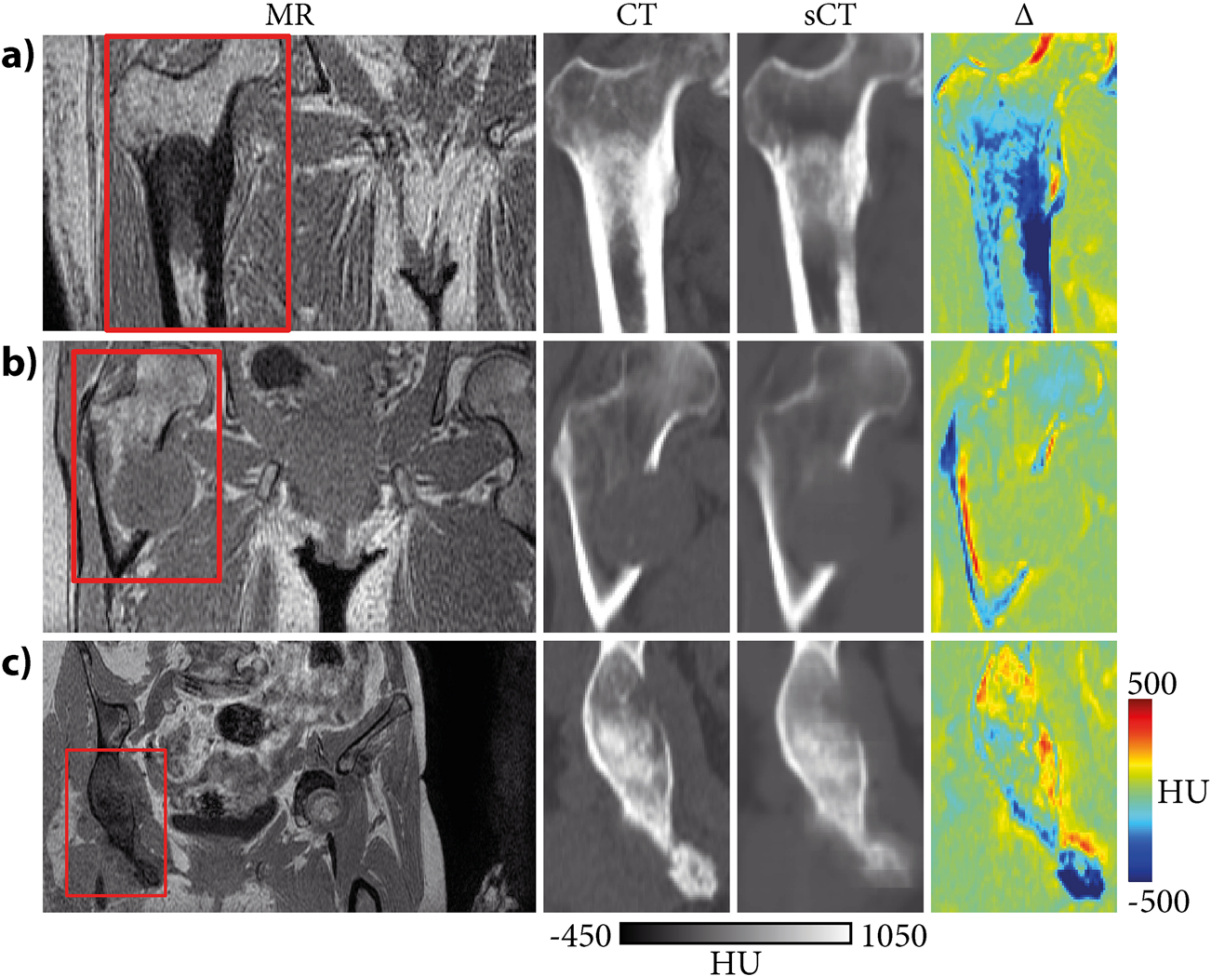
MR, CT, synthetic CT (sCT), and sCT-to-CT difference (Δ) obtained for three patients presenting (**a**) osteoblastic, (**b**) osteolytic, and (**c**) mixed lesions. Red boxes on the MR images indicate the region that was zoomed in to compare CT and sCT images

Representative cases of sCT images were obtained for patients presenting osteoblastic (Fig. 2a), osteolytic (Fig. 2b), and mixed (Fig. 2c) lesions along with sCT-to-CT difference maps. The soft tissues around the lesions were well characterized in MR images. Most errors were located in dense and sclerotic regions, although bone formation was identifiable. Synthetic CT images were slightly blurred causing some of the intensity underestimation seen in Tables 3 and 4.

**Fig. 3:**
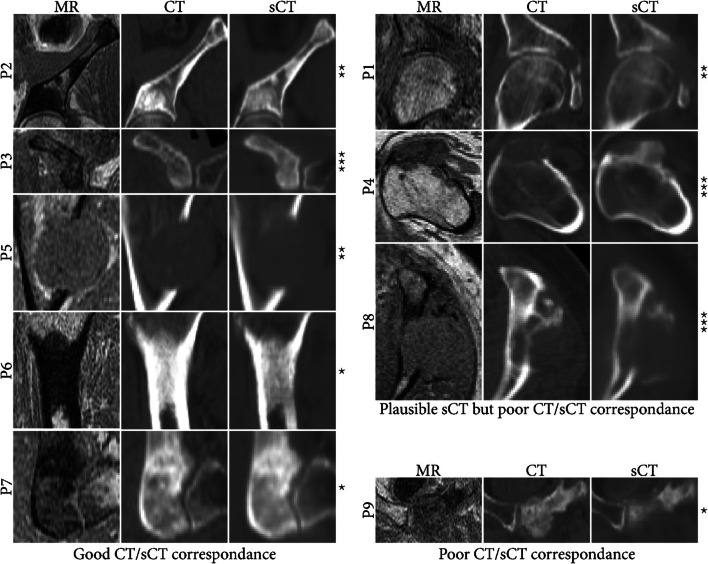
Comparison of single slices out of the MR, CT, and sCT datasets of all patients, divided by their sCT-CT correspondence in the lesion. For most patients, lesions could be correctly identified on sCT images. For three patients, differences were observed between CT and sCT images, but it is hard to judge whether they are due to pathological changes (e.g., calcium-enriched bone visible in CT but not MRI in P8) or error in sCT reconstruction. For one patient, definite sCT reconstruction errors are visible with sclerotic regions not well depicted. Stars indicate different time spans between pretreatment CT and treatment MR: * < 10 days, ** 10–35 days, *** > 100 days

Overall, a good sCT-CT correspondence has been observed as shown Fig. 3 which displays the sCT images of all patients. For three patients, significant differences between sCT and CT were visible, but their cause, sCT generation errors or interscan pathological changes, can hardly be determined given the long timespan between the MRI and CT acquisitions. For one patient, the model largely underestimated the sclerotic region.

**Fig. 4:**
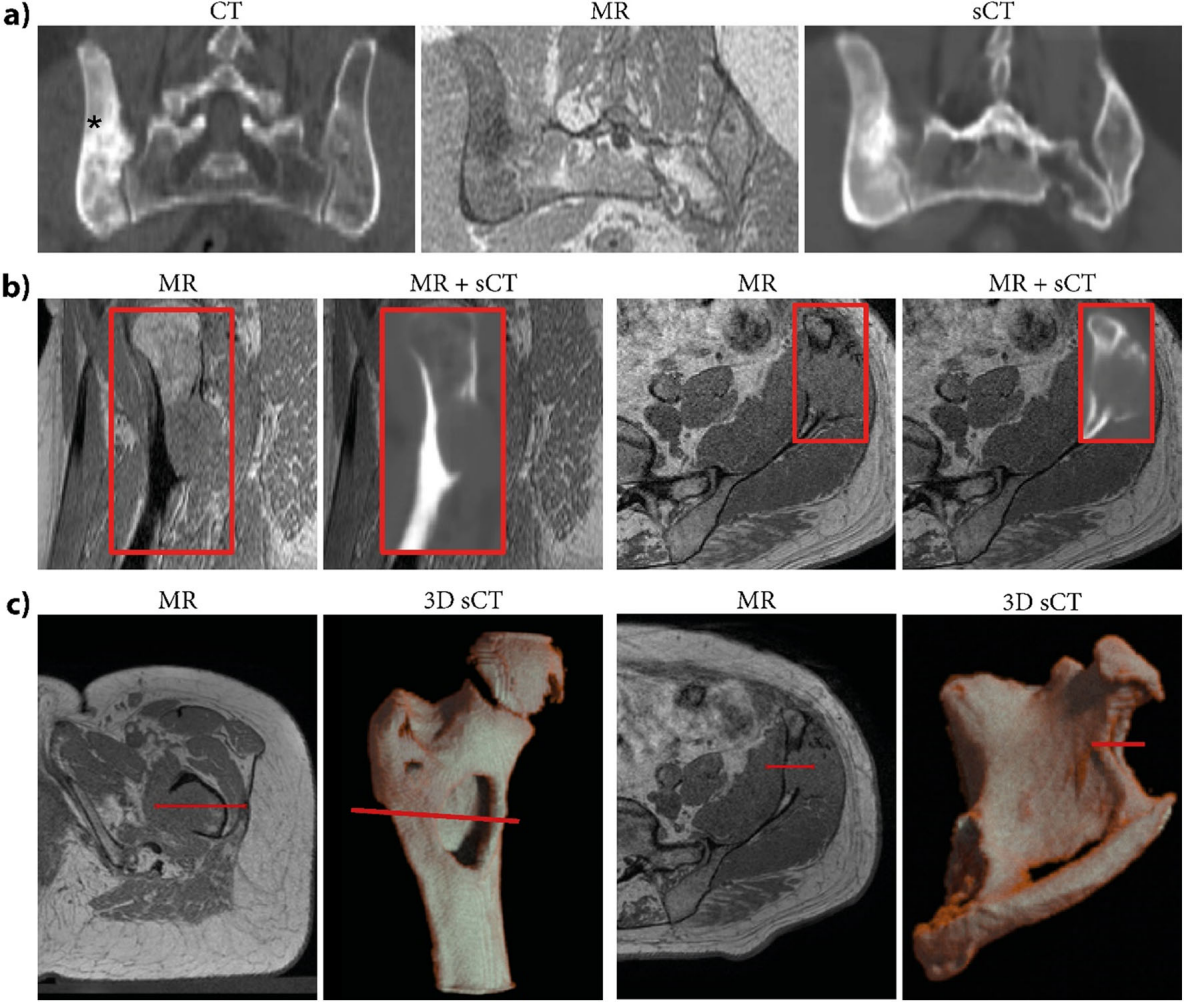
**a** Pretreatment CT images in the pelvis with conventional patient positioning and MR and intrinsically aligned synthetic CT (sCT) images with unconventional patient positioning for the HIFU treatment. Lesion is indicated by *. Since sCT scans are intrinsically registered with MR, they are able to provide bone cortex depiction with the patient in treatment position. **b** MR and a fused visualization of MR and sCT images depicting the cortical bone distribution, obtained for two patients with osteolytic lesions in the femur (left) and in the pelvic iliac crest (right). MR and sCT are inherently registered, allowing straightforward identification of soft tissues and bone. **c** MR images from transverse slices of two patients with lesions in the femur and pelvis and corresponding 3D bone renderings that provide an overview of the bone with the lesion. The bone renderings were obtained by thresholding the sCT at 150 Hounsfield units within the bone mask (created in MeVisLab v3.2, MeVis Medical Solutions AG). The red line identifies the same location in MR images and 3D bone renderings

Figure 4 shows the potential use of sCT as a bone visualization tool for planning of MR-HIFU treatment in the pelvis and femur.

## Discussion

In this study, we evaluated the potential of deep learning–based synthetic CT generation for the visualization of cancellous and cortical bone to support MR-HIFU treatment planning for metastases in pelvic and femoral bones. Combining MRI and CT-like imaging in a single reference frame would be useful to better define the region of treatment and the bone cortex, during treatment planning for pain palliation (and tumor ablation, if attempted). The short sCT generation time (< 2 min) required per patient makes this approach suitable for on-the-fly treatment planning. Pretreatment CT and sCT images showed similar cortical bone distribution in patients with bone metastases.

For treatment planning, qualitative visualization of the bone distribution in the lesion and its vicinity is paramount. Inadequate depiction of the lesion can result in over- or undertreatment of the lesion and potential damage to the surrounding tissue. CT could be used for bone cortex depiction, but a recent CT is not always available and large differences in patient positioning during pretreatment CT imaging and treatment MR imaging are often present. Synthetic CT offers an easier interpretation of the bone distribution in treatment position, facilitating physicians to perform the ablations [9]. The delineation and characterization of the lesion could be performed on MRI. However, sometimes, it is unclear where remaining parts of cortical bone are present, solely based on (intraprocedural) MRI. Here, sCT might have additional value for characterization and/or delineation of the metastasis. Moreover, sCT images could potentially be used for identification of other bony structures along the US beam path and serve as input for sonication simulations to facilitate treatment cell positioning and optimization of sonication protocols for energy deposition in the target lesion [33, 34]. This approach of combined sCT and MR images could also be of value for other fields of MR-HIFU applications in bones, such as treatment of osteoid osteoma. However, the network will probably need to be re-trained with osteoid osteoma data.

The quantitative metrics obtained in this study are comparable to the results reported in literature, with MAD in the bone larger than 125 HU reported in the lower arm and pelvis for patients with no known orthopedic conditions [16, 27]. However, this observation on limited data does not allow drawing general conclusions.

In its current implementation, the model was able to provide a rough estimation of the cortical bone distribution in and around the lesion. The exact lesion contour was more approximate, presumably because of its irregular geometry, which might cause partial volume issues in images, and of the slightly blurred sCT images.

Anatomical changes caused by tumor progression could partially explain the lower DSC and higher RMSD of the surface distance between CT and sCT in the lesion. For 6/9 patients, the timespan between the CT and MR was longer than 10 days, the approximate time interval in which anatomical changes would typically occur (Fig. 2 and example case in Supplementary Materials, Figure S2). Motion artifacts could also afflict the sCT generation and its comparison with CT (see P1 in Supplementary Materials, Figure S3). For one patient, definite sCT reconstruction errors were observed, presumably because the metastasis was newly formed as evidenced by hyperintense signal in the almost opposed-phase image and hypointense signal in the almost in-phase image [35]. This hyperintense signal was observed only in this patient (Supplementary Materials, Figure S4), and the sCT error could be solved either on the processing side, by adding more newly formed osteoid cases to the training set, or on the acquisition side, by acquiring MR data with more than 2 echoes, to make the model less sensitive to the relaxation time T2* (shorter in this lesion compared to other tissues).

We acknowledge several limitations to this study. First, the presented results show the potential of MR-based synthetic CT for a limited dataset and more data are required to assess the robustness of the method across patients. However, the lesions were all located in the hip region, which is the most commonly treated region with MRI-HIFU [5]. To limit overfitting caused by the small dataset, a patch-based method with a limited receptive field was used to facilitate the generalizability of the model and data augmentation was applied to feed the network with data corresponding to unconventional patient positioning.

In addition, only the metastatic bone and the lesion were evaluated with sCT, as soft tissues can be better assessed on MR images. Bone reconstruction was slightly blurry, partly because of registration errors in the training set, mainly due to positioning differences between pretreatment and treatment scans [36]. In the future, by acquiring pre-treatment MR scans in addition to the pre-treatment CT scans, the registration of the training set would be improved and interscan differences would be reduced, leading to the generation of sharper sCT images.

To conclude, this study demonstrated the potential of sCT for visualizing cancellous and cortical bone distribution for HIFU treatment procedures of bone metastases in the hip region. Osteolysis and ossification were visible on the sCT images with the bone distribution comparable between CT and sCT within the bone of interest. Thus, synthetic CT images could help in visualizing bone lesions with CT-like contrasts for planning of palliative MRI-HIFU procedures, including the targeting of the lesions and treatment planning.

## Supplementary Information

ESM 1 (DOCX 1428 kb)

## Abbreviations

DSC: Dice similarity coefficient
HU: Hounsfield unit
MAD: Mean absolute difference
MD: Mean difference
MRI-HIFU: Magnetic resonance imaging–guided high-intensity focused ultrasound
RMSD: Root mean square distance
sCT: Synthetic CT

## Declarations

### Guarantor

The scientific guarantor of this publication is Lambertus W. Bartels.

### Statistics and Biometry

No complex statistical methods were necessary for this paper.

### Methodology

• retrospective

• experimental

• performed at one institution
