# Supplementary material for: Synthetic CT for the planning of MR-HIFU treatment of bone metastases in pelvic and femoral bones: a feasibility study
Source: Eur Radiol. 2022 Feb 21;32(7):4537–46. doi: 10.1007/s00330-022-08568-y (PMC9213310; doi:10.1007/s00330-022-08568-y)
Supplement: Supplementary file 1 — (DOCX 1428 kb) [file 330_2022_8568_MOESM1_ESM.docx]

**Supplementary Materials**

S1.


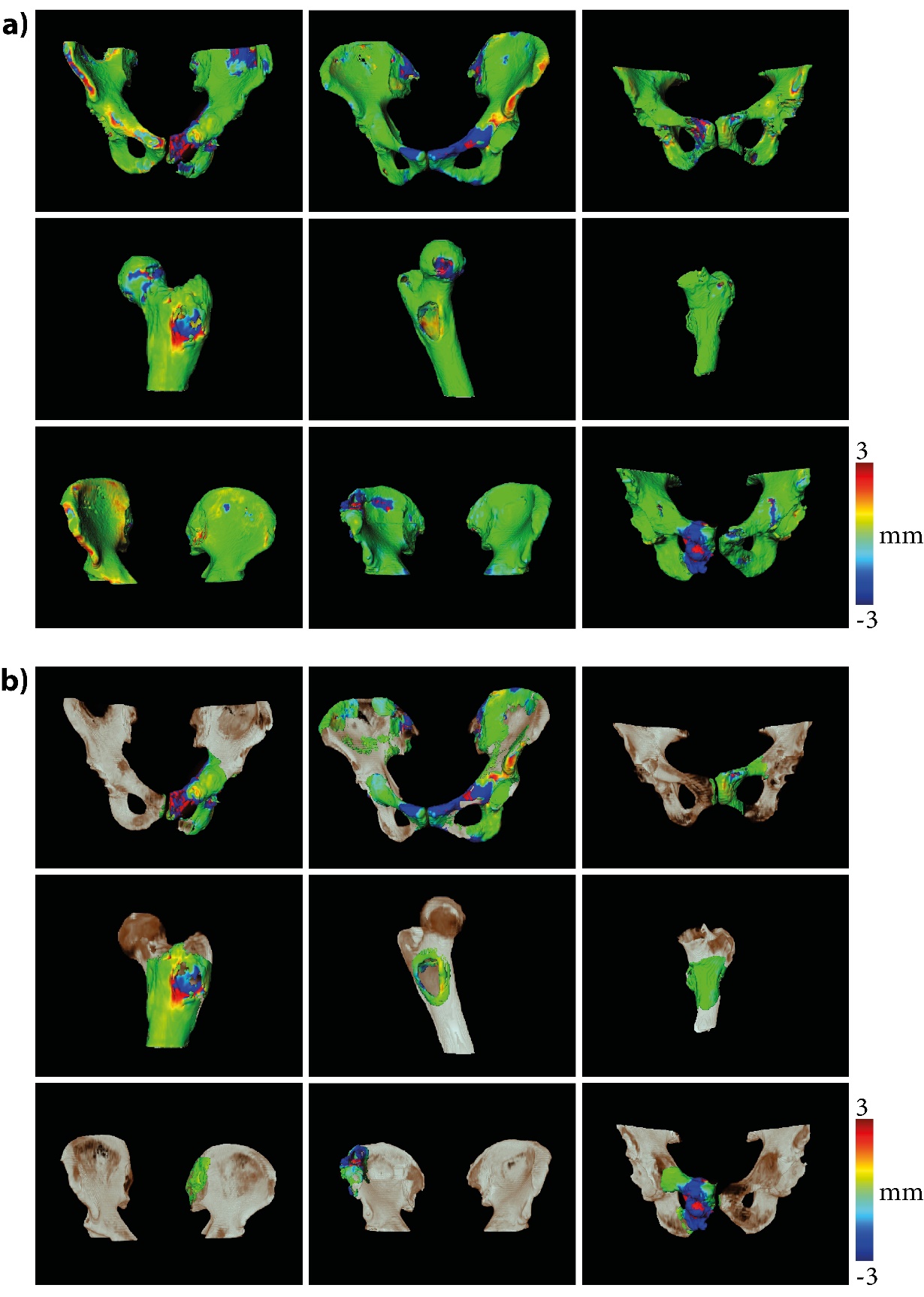


*Figure S1. sCT-to-CT surface distance maps of the patients images obtained in this study. (a) in the entire bone, (b) on a 3 cm margin around and including the lesion.*

S2. Possible changes in the lesion between the acquisitions are problematic when comparing sCT (generated from treatment MR) with pretreatment CT. For example, for patient 8, such changes were documented on two subsequent CT scans acquired 2 months apart, followed by MR HIFU treatment another 2 months later. Pathophysiological changes were observed between March and May and so could be expected between May and July. Example of CT scans from patient 8:

**CT – 21 March 2019 CT – 15 May 2019**


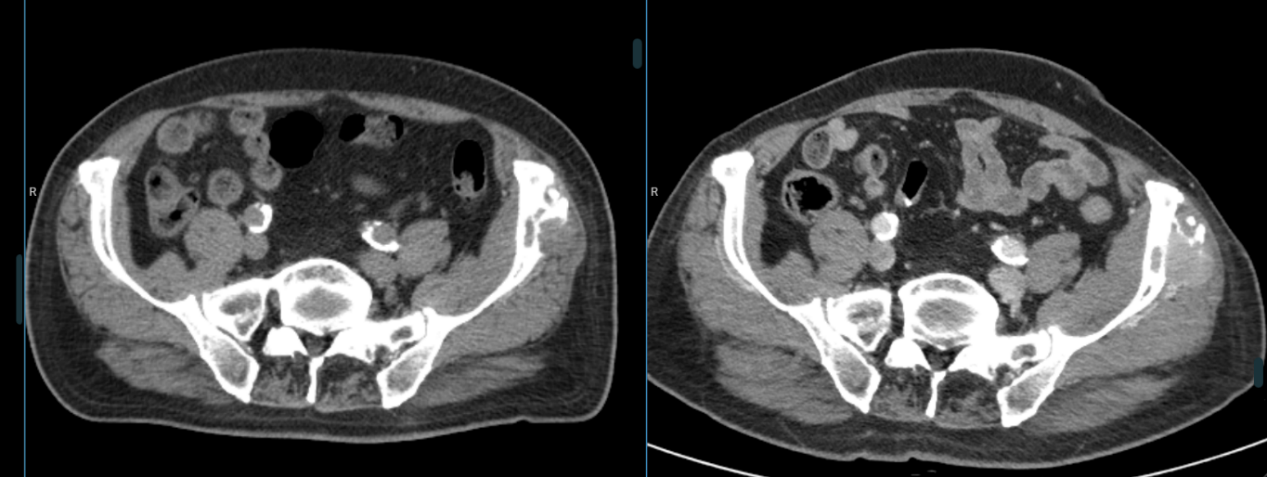


*Figure S2. The osteolytic progress of the lesion between 2 CT scans, leading to local changes close by and in the pelvis bone. The lesion is indicated by a red square. The CT scan acquired in May was contrast-enhanced.*

S3. An MR image afflicted by motion artefacts could result in sCT of lower quality, especially when compared to a CT scan. This could explain the low DSC achieved in the lesion in P1. Example of pretreatment MR from patient 1:


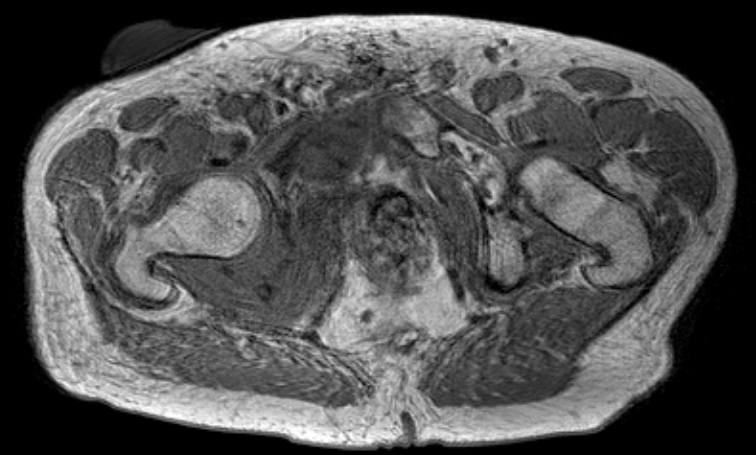


*Figure S3. Example of pretreatment MR image with motion artefacts (P1). The lesion is indicated by a red square.*

S4. From Figure 2, the metastatic tissue is not recognized as osteolytic by the network, but it is instead reconstructed as soft tissue. It has been shown that osteoblastic lesions appear hypointense on the T1- and T2-weighted MRI scans [37] and that they could show signal loss in opposed-phase images[35]. This behavior of sclerosis on MRI may be explained by the short T2 relaxation time due to dense mineralization of the sclerotic area. This appearance has been observed in the osteoblastic patients in this study. However, in P1, the lesion in the almost opposed phase (TE=2.1 ms) doesn’t show signal loss. This could be explained as in newly formed osteoid, water and fibro vascular tissue predominate when compared to mature sclerosis.


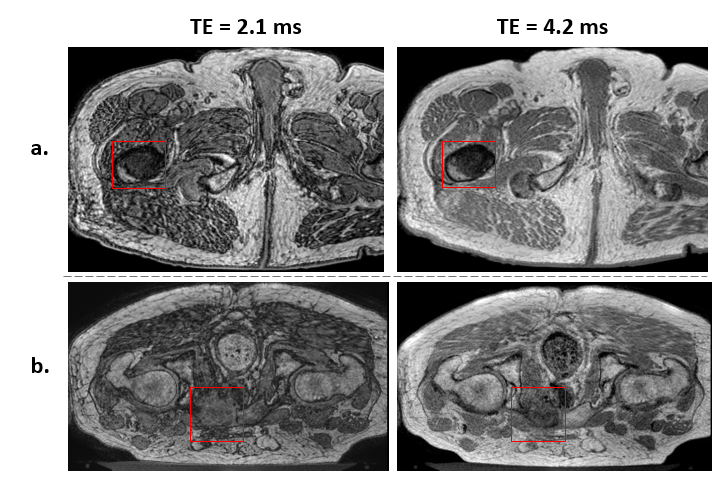


*Figure S4. Example of a. almost in-phase and almost opposed -phase image of most osteoblastic lesions present in the dataset and of b. almost in-phase and almost opposed -phase image for P9. The lesion is indicated by a red square.*
